# Risk-adapted partial larynx and/or carotid artery sparing modulated radiation therapy of glottic cancer

**DOI:** 10.1186/1748-717X-9-136

**Published:** 2014-06-13

**Authors:** Stefan Janssen, Christoph Glanzmann, Gerhard Huber, Gabriela Studer

**Affiliations:** 1 Department of Radiation Oncology, University Hospital Zurich, Rämistrasse 100, Zürich CH-8091, Switzerland; 2 Department of Otorhinolaryngology, Head and Neck Surgery, University Hospital Zurich, Zurich, Switzerland

**Keywords:** Glottic cancer, IMRT, Partial larynx sparing, Carotid sparing

## Abstract

**Background:**

To evaluate outcome in patients with glottic cancer treated with intensity-modulated radiotherapy (IMRT) and to show effectiveness of partial laryngeal- and/or carotid artery sparing in low to intermediate risk tumors.

**Study design:**

Retrospective analysis.

**Material and methods:**

From 01/2004 to 03/2013 77 consecutive patients presenting with glottic cancer were treated in our department with IMRT as definitive treatment. T-stages distributed as follows: T1: n = 17, T2: n = 24, T3: n = 15, T4: n = 13 and recurrences: 8 patients. Concomitant systemic therapy was applied in 39 patients consisting of either cisplatin or cetuximab.

**Results:**

Mean/median follow-up (FU) time was 32.2/28 months (range: 4–98.7). Three year local control (LC), ultimate LRC and laryngectomy free survival rate was 77%, 92% and 80%, respectively. Three year overall survival of the entire cohort was 81%. Three year local control for T1/T2, T3/T4, and recurred tumors was 95%, 65%, and 38%, respectively. Three year overall survival was 86% for T1-4 stages, 55% for recurred disease, respectively. Partial laryngeal/carotid artery sparing was performed in all T1 patients (n = 17) and 17/22 T2N0 patients. Rate of late sequels was low.

**Conclusion:**

IMRT for glottic cancer shows high control rates. In low to intermediate risk tumors an individualized treatment volume with partial larynx +/- carotid artery sparing is effective and holds the potential to reduce long term toxicity. The therapeutic outcome was not compromised.

## Background

Glottic cancer is the most common laryngeal cancer and no standard treatment has been established. Early glottic cancer (T1-2 N0) is often treated with local radiotherapy (RT) alone showing similar local control rates of 80-90% compared to partial laryngectomy/laser resection [1]. Accelerated schedules of 63 Gy in 28 fractions 5 times a week or 68/62 Gy in 34/32 fractions 6 times per week seem to beneficial [2,3]. In advanced laryngeal cancer larynx preservation is aimed with RT in combination with systemic therapy (as induction and/or concomitant) [4,5]. More advanced disease with consecutive laryngeal dysfunction often requires a laryngectomy followed by radio-/chemotherapy.

Against the background of the above mentioned excellent control rates in early stage laryngeal cancer, chronic sequels gain importance. Radiation-induced carotid artery disease is long known [6]. In past years several series reported on radiation-induced arteriosclerosis [7], stenosis [8-11], and consecutive ischemic stroke [12-14]. In conventional opposed fields the carotid arteries often receive full dose. Intensity-modulated radiotherapy (IMRT) offers the potential to spare surrounding tissues, e.g. carotid arteries or parts of the larynx [15-21]. Latter series only include few patients or consist of dosimetric comparison of different treatment techniques only.

We present control rates of the, to our knowledge, largest patient collective of laryngeal cancer patients treated with carotid artery and/or partial larynx sparing IMRT in low and intermediate stages.

## Material and methods

### Patients

From 01/2004 to 03/2013 77 consecutive patients presenting with glottic cancer were treated in our department with IMRT as definitive treatment. Eight patients presented with a local recurrence after trans-oral laser resection (10%). A pretreatment panendoscopy and computed tomography (CT) was carried out before treatment providing anatomical information for delineation of the planning target volume (PTV). Diagnosis was proven histologically in all patients showing squamous cell carcinoma (SCC). Patient and treatment related parameters are summarized in Table 1. In six patients a tracheostomy was carried out before RT start (8%).

**Table 1:** Patient and treatment related parameters

Gender	
Male	68 (88%)
Female	9 (12%)
Mean age (years, range)	67 (35–87)
Mean gross primary tumor volume (pGTV, ml, range)	9.5 (0.2–88.7)
Planning target volume (PTV, ml, range))	76.2 (18–283)
T-stage	
T1	17 (22%)
T2	24 (31%)
T3	15 (20%)
T4	13 (17%)
Recurrence	8 (10%)
N-stage	
N0	63 (79%)
N1	4 (5%)
N2a	0
N2b	5 (8%)
N2c	5 (8%)
N3	0
Concomitant systemic therapy	39 (51%)
Cisplatin	26 (34%)
Cetuximab	14 (18%)
Both (sequentially n = 2, simultaneously n = 1)	3 (4%)
RT prescription dose (total/single)	
61.6/2.2 Gy	1 (1%)
63/2.25 Gy	2 (3%)
69.6/2.11 Gy	7 (9%)
66/2 Gy (6 f/week)	10 (13%)
68/2 Gy (6 f/week)	25 (32%)
70/2 Gy	32 (42%)

Regular follow-up (FU) visits were carried out in our joint clinic at the Department of Otorhinolaryngology, Head and Neck Surgery. Institutional standards for patient assessment included physical examination and flexible fiber optic endoscopy approximately every 2 months in the first year of FU, every three months in the second to third year and every 6 months in the fourth to fifth year.

Analysis was approved by the ethics committee of the Zurich university hospital.

### IMRT/VMAT

We used simultaneously integrated boost (SIB) technique in all patients. SIB-IMRT technique was performed using the following schedules:

SIB2.00: 34–35 fractions with daily SIB doses of 2.00 Gy (PTV1)/1.70 Gy (PTV2) and 1.54 Gy (PTV3) to a total boost dose of 66.00-70.00 Gy (six/five fractions a week).

SIB2.11: 33 fractions with daily SIB doses of 2.11 (PTV1)/1.80 Gy (PTV 2) and 1.64 Gy (PTV3) to a total boost dose of 69.60 Gy (five fractions a week).

The dose was normalized to the mean dose in PTV1. For intensity optimization, the prescribed dose encompassed at least 95% of the PTV. Additionally, no more than 2% of any PTV received >110% of its prescribed dose.

Target volumes were delineated as follows: The primary and involved lymph nodes included the gross extent of disease, taking clinical and radiological findings into account; CTV was defined by adding 10-15 mm margin to the GTV, another 2-3 mm margin was added from CTV to PTV 1 dependent on proximity to critical structures; PTV2 covered areas considered at high risk for potential microscopic disease; and PTV3 included lymphatic pathways (elective PTV coverage). If possible we tried to spare contralateral vocal cord and hypopharyngeal region from high dose volume.

To ensure sufficient dose delivery to the skin close to GTVs, bolus material (0.5-1 cm thickness) was used in all patients with <5 mm between GTV and the overlying skin as well as involvement of the anterior commissure (n = 70, 91%). Hot spots in the arytenoid cartilage were avoided.

Irradiation was delivered with four to five coplanar beam angles by a 6-MV dynamic MLC system (Varian Medical Systems, Palo Alto, CA) using sliding window technique, or using volumetric modulated rapid arc technique (VMAT, since 04/2010 (n = 13)). Patients were immobilized from head to shoulders using a commercially available thermoplastic mask in supine position.

Keeping the possibility of anatomical miss mentioned by Osman et al. in mind, good quality assurance is mandatory [22]. We advise our patients not to swallow during treatment. In addition daily kilovolt (KV) imagines and regularly cone-beam computed tomographies (CB-CT) (also to control the position of bolus material) are carried out. Aberrations of more than 2 mm are corrected before daily irradiation.

### Treatment volumes

In T1 tumors and favorable T2 tumors without bulky disease, no elective lymph node irradiation was carried out (T1: 17/17, T2N0: 4/22). High dose planning treatment volume was restricted to the involved area of the larynx in order to spare contralateral laryngeal structures and carotid artery (T1: in 15/17, T2N0: in 16/22, T3: in 10/15). In those patients carotid arteries were delineated on planning CT on both sides (excluding patients with elective node irradiation (ENI)).

Mean primary gross tumor volume (pGTV) was 9.5 ccm (range: 0.2-88.7). Mean volume of planning target volume (PTV) was 76.2 ccm (range: 18–283). For T1 tumors mean GTV and PTV was 1.2 ccm (range: 0.2-3.5) and 40.9 ccm (range: 18–92), for T2N0, the corresponding mean GTV and PTV was 2.9 ccm (range: 0.7-7.6), and 55.7 ccm (range: 35–123), respectively.

### Systemic therapy

In case of recurrent tumor after laser resection, local advanced T-stage, positive lymph nodes and bulky T2 tumors, systemic therapy was aimed. Systemic therapy preferably consisted of cisplatin (40 mg/m^2^ weekly) and was switched to cetuximab in case of cisplatin related adverse effects (cetuximab loading dose: 400 mg/m^2^ followed by weekly applications of 250 mg/m^2^). For patients with contraindications against cisplatin, cetuximab was favored primarily. Age, Karnofsky performance score and comorbidities were respected.

In our study systemic therapy was carried out in 38 patients either with cisplatin (n = 21) or cetuximab (n = 14) or combination of both (switch after cisplatin related side effects (n = 2) or within a clinical trial applying concomitant cisplatin and cetuximab (n = 1)). Cisplatin had to be stopped due to pancytopenia after 1 (n = 1) or 3 courses (n = 1), or due to rising level of creatinine after 2 (n = 1) or 3 courses (n = 1) or due to nausea after 2 (n = 1) or 4 courses (n = 1). Cetuximab had to be stopped due to reduction of general condition after 3 courses (n = 1) and skin toxicity after 3 courses (n = 1).

### Statistics

Statistical calculation was performed using the statistic program implemented in StatView (Version 4.5; SAS Institute, Cary, NC).

## Results

### Disease control

Mean/median follow-up was 32.2/28 months (range: 4–98.7). By the time of analysis 19 patients were dead (25%). Three of them suffered from tumor progression, 10 died due to other causes (heart attack (n = 4), pneumonitis (n = 1), stroke (n = 1), pulmonary embolism (n = 2), sepsis (n = 1), progressive thyroid cancer (n = 1)). In the remaining 6 patients the cause of non-disease related death is not known because they were lost in follow-up.

**Figure 1:**
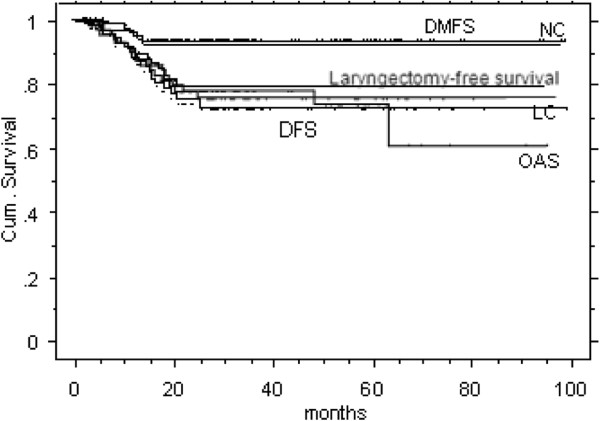
**Kaplan Meier curves for the entire study cohort.** DMFS = distant metastases free survival, NC: neck control, LC = local control, DFS = disease free survival, OAS = overall survival.

Four patients developed distant metastases (lung: (n = 4), mediastinal lymph nodes (n = 1)). Two patients had persistent nodal (and primary) disease; one patient newly developed cervical lymph node metastases. In that case a neck dissection was carried out in curative intention. We observed 13 local failures (including 2 patients with persistent disease) (17%).Radical laryngectomy was carried out as salvage therapy in all 13 patients (after a mean time period of 11 months after completion of RT (range: 3–18)). The 3 year ultimate local-regional control rate was 92% (ultimate control: outcome in patients with salvage surgery after local or nodal failure included). Local control, nodal control and laryngectomy free survival after 3 years was 77%, 93% and 80%, respectively (Figure 1), overall survival was 81%. Local control for T1/T2, T3/T4, and recurred tumors was 95%, 65%, and 38%, respectively. Overall survival was 86% for T1-4 stages, 55% after IMRT of recurred disease, respectively.

### Partial larynx sparing

Partial larynx sparing IMRT was carried out in 43/54 T1-3 N0 tumors (treatment volumes: Table 2). In two T1N0 and one T2N0 partial larynx sparing was not possible due to tumor location close to the midline. In more advanced tumors partial larynx sparing was not aimed.

**Table 2:** Simplified treatment volumes used in patients with T1-T4 laryngeal carcinomas

	**PTV1 (64–70 Gy)**	**PTV2 (60 Gy)**	**PTV3 (54 Gy)**
T1	Affected laryngeal side (~1/2-2/3 of laryngeal volume)	Small region above and below PTV1	–
T2	Affected laryngeal side (~2/3 of laryngeal volume)	Small region above and below PTV1	Level II-V bilaterally (for small T2 tumors no ENI)
T3	Most of the larynx depending on tumor extension (~3/4- 4/5 –entire laryngeal volume)	Small region above and below PTV1	Level II-V bilaterally
T4	Whole larynx	Small region above and below PTV1	Level II-VI bilaterally
N+	Affected lymph node sites	Small region around PTV1	Level II-VI bilaterally

*ENI* elective node irradiation.

### Carotid artery sparing

In all 17 T1N0 patients and four T2N0 patients bilaterally carotid sparing was performed. In cases of lymph node metastases or ENI no carotid sparing could be performed.

The mean dose to the ipsilateral contoured segment of the carotid artery was 29.4 Gy (range: 1.1-72). The mean dose to the contralateral carotid was 20.2 Gy (range: 1–54.1). The mean volume receiving more than 35 Gy (V35), 50 Gy (V50) and 63 Gy (V63) was 30.1%, 8.0% and 2.0% for the ipsilateral, and 7.1%, 0.5%, and 0% for the contralateral carotid artery, respectively.

### Tolerance

Rate of late sequels was low. 16 patients were in need of a temporary gastric tube (21%, T1: n = 1, T2: n = 3, T3: n = 3, T4: n = 8, recurrence: n = 1). One patient (cT4c2b) developed a CTCAE grade 4 laryngeal edema during the last week of radio-chemotherapy and was still in need of a gastric tube at time of last FU visit (10 months after therapy: alive and no evidence of disease). No other ≥ grade 3 adverse effects were observed.

## Discussion

Radiotherapy is a major component in glottic cancer therapy. In early stages it offers equal tumor control compared to surgery. In advanced stages radio-chemotherapy holds the chance for a conservative treatment approach in order to avoid laryngectomy.

We could show that partial larynx sparing and/or carotid artery sparing is feasible in most low to intermediate risk glottic cancer patients without compromising tumor control.

### Larynx sparing IMRT

**Figure 2:**
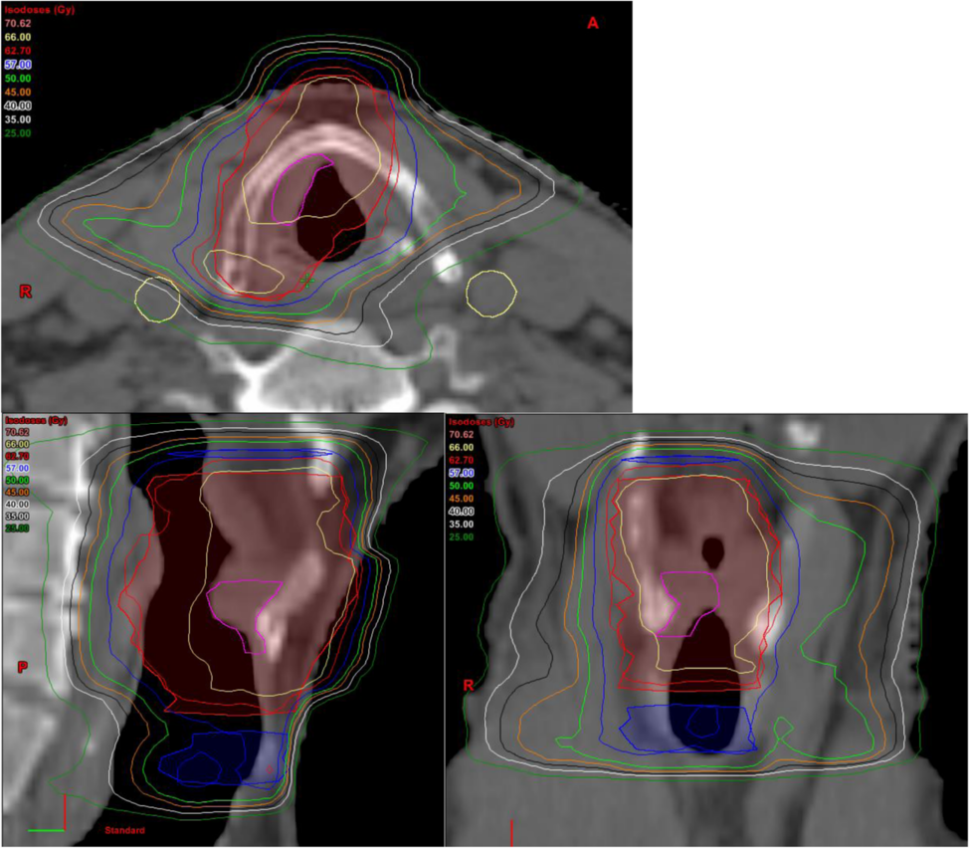
Treatment plan of a patient with a T1N0 glottic carcinoma with high dose coverage of involved side only, red: larynx sparing PTV1: 66 Gy, blue: PTV2 (60 Gy), yellow: carotid arteries.

**Figure 3:**
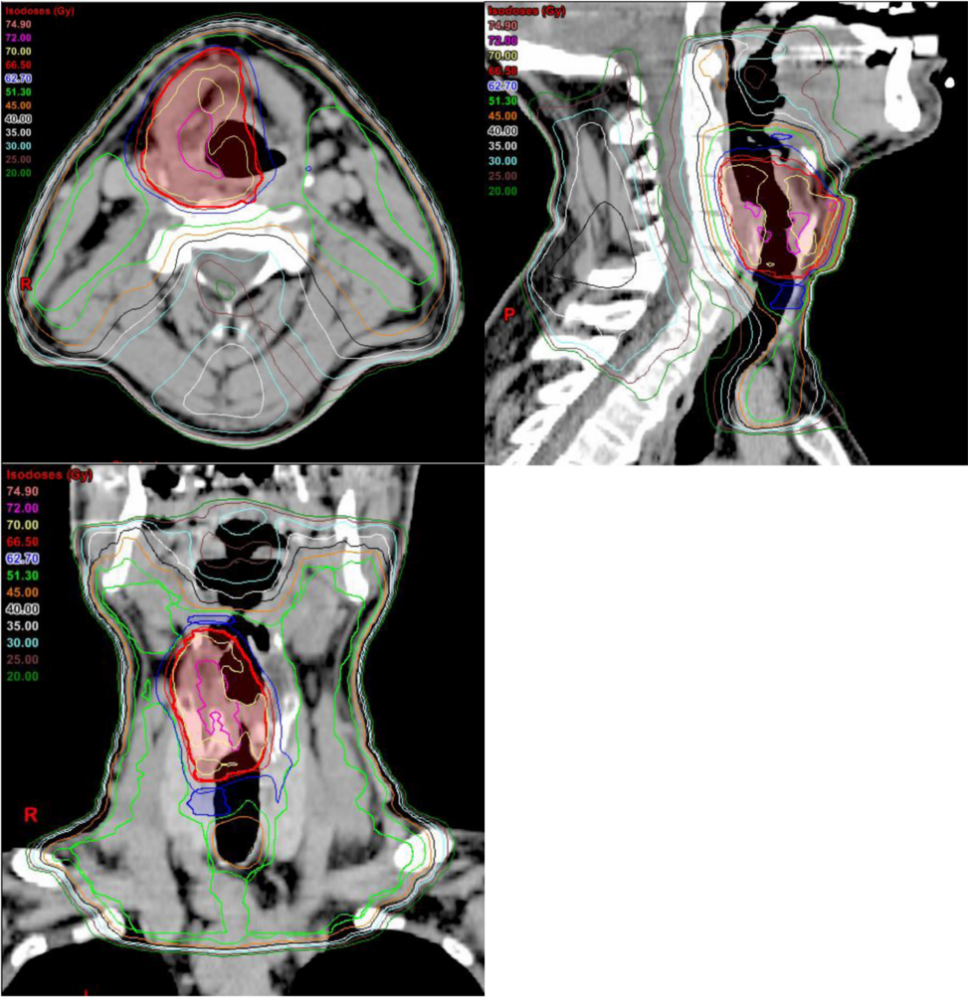
Example of treatment plan of a patient with a T3N0 glottic carcinoma, red: larynx sparing PTV1 (70 Gy), blue: PTV2 (60 Gy), green: PTV3 (54 Gy), pink: GTV.

In advanced tumor stages it is recommended to include the larynx generously in treatment volume. Additionally, bilateral level II-IV lymph node regions are treated. In the clinically node negative neck Eisbruch et al. advise to include only the subdigastric nodes as upper border of level II, for the clinically involved side the upper border should reach the base of the skull reference. For stage T2 the risk of lymph node metastases is borderline [23-25]. In advanced T2 disease (with reduced vocal cord mobility) elective irradiation of the neck (ENI) may be indicated [26]. In our patient collective we included level II-IV bilaterally in stage T2N0 disease by the majority. Only small T2N0 tumors were treated without ENI (n = 4/22). For small tumors we reduced the high dose volume to the affected area in order to spare contralateral laryngeal structures. This was realized in most T1-T3N0 tumors (43/54, Figures 2 and 3). Partial larynx sparing is expected to reduce long term toxicity, which has been shown low in our cohort. Haderlein et al. recently stated that the dose to anatomical structures responsible for swallowing function appears to play a role in the treatment of laryngeal cancer [27]. Treatment volumes for different tumor stages are summarized in Table 2.

In recent years several study groups showed IMRT to be effective in laryngeal cancer with comparable control rates and improved tolerance compared to conventional techniques ( [28-32], see Table 3).

**Table 3:** IMRT studies in larynx tumors

	**Patients (n)**	**Tumor stage**	**Chemotherapy**	**LRC**	**OS**
Eisbruch [30]	11	n.a.	n. a.	60% (3 yrs)	n. a.
Yao [29]	33	n.a.	n. a.	85% (2 yrs)	n. a.
Lee [31]	20	III/IV: 100%	100%	90% (2 yrs)	69% (2 yrs)
Studer [28]*	58	I/II: 31%	85%	65% (3 yrs)	78% (3 yrs)
		II/IVA: 69%			
Nguyen [32]	8	III/IVA: 100%	100%	85.2% (2 yrs)	n. a.
Current study	77	I/II: 54%	28%	76% (3 yrs)	81% (3 yrs)
		II/IVA: 46%	79%		

*LCR* loco-regional control, *OS* overall survival, *n. a.* not applicable for laryngeal cancer exclusively.

*small number of patients included in both series.

For early stage disease carotid sparing IMRT was tested (mainly as comparative planning studies based on small samples) few years ago by several investigators (Table 4). In 2010 Rosenthal et al. from M.D. Anderson Cancer Center revealed significantly reduced radiation dose to the carotid arteries compared with conventional lateral fields while maintaining target volume coverage [20]. Other study groups showed similar results [21,33]. Chera and Feigenberg et al. challenge the interpretation of a new standard because of the risk of complication from dose heterogeneity [15,34].

**Table 4:** Carotid-sparing IMRT studies in early laryngeal cancer

	**Patients (n=)**	**Tumor stage**	**Prescription total/single dose (Gy)**	**Objective**	**Results**
Rosenthal [20]	11	T1-2 N0	65/2.25	Comparison of opposed lateral fields and IMRT	Best carotid-sparing with IMRT
Chera [15]	5	T1N0	63/2.25	Comparison of opposed lateral field, 3D-RT and IMRT	Best carotid-sparing with IMRT
Sert [16]	5	T1N0	62.25/2.25	Comparison of opposed lateral field, 3D-RT and IMRT	Best carotid-sparing with IMRT (V35, V50, V63), identical conformity
Atalar [17]	5	T1N0	63/2.25	Comparison of conformal RT, IMRT and IMAT	More hot spots in IMRT and IMAT, less dose to carotids with IMRT/IMAT
Osman [21]	0 (comparative planning in 10 cases)	T1N0	66/2	Comparison of conventional plans and IMRT (coplanar and non-coplanar)	Contralateral vocal cord sparing best with single vocal cord RT IMRT
Mourad [19]	0 (comparative planning in 1 case)	T1N0	63/2.25	Comparison of 2D, 3D and IMRT plans in a patient with complete left carotid artery occlusion	Minimal dose to right carotid artery and pharyngeal constrictor with IMRT
Riegel [18]	0 (comparative planning in 11 cases)	T1-2 N0	63/2.25	Comparison of lateral opposed fields, VMAT (full-arc, half arc) and IMRT	Full-arc VMAT offers best carotid sparing (and highest mean normal tissue dose), static IMRT produced next-best carotid sparing

*IMRT* intensity-modulated radiotherapy, *IMAT* intensity-modulated arc therapy, *VMAT* volumetric-modulated arc therapy.

To our knowledge the here presented series includes the largest number of glottic tumor patients treated with carotid sparing IMRT (n = 21) including a FU of mean/median 32.2/28 years. The 3 years control rates are comparable with historic series using conventional techniques [35].

### Carotid sparing IMRT

The risk of carotid artery stenosis after RT and consecutive ischemic stroke is well known [7-14]. However, there is no knowledge available regarding the influence of the applied dose to the artery, length of irradiated vessel, patients’ age or pre-existing vascular variances on vascular changes. Especially in patients with long life expectation after curative treatment this has to be taken into account. Additionally patients with pre-existing vascular risk factors (smoking history, diabetes, and hypercholesterolemia) may benefit.

In early glottic cancer patients, we were able to keep mean doses to carotid arteries below 25 Gy. Martin et al. reported vessel wall abnormalities only at doses ≥35 Gy or ≥50 Gy. In our patients with carotid sparing IMRT, vessel volume receiving more than 35 Gy (V35) and V50 was below 20% and 5% of the contoured segments, respectively [10]. Carotid artery sparing is principally known realizable also with conventional radiation techniques, by using IMRT this was found technically easily feasible in most T1-2 patients. A clinical benefit could not be quantified based on this study set up. As a limitation of the present study one has to state that carotid sparing could not be realized in patients with ENI.

## Conclusion

Definitive partial larynx +/- carotid artery sparing IMRT with highly conformal boost PTV is effective without compromised control rates. A longer FU is needed to strengthen the hypothesis of an improved therapeutic ratio.

### Consent

Written informed consent was obtained from the patient for the publication of this report and any accompanying images.

## Competing interests

On behalf of all authors, the corresponding author states that there is no conflict of interest. Financial disclose: No financial or material support.

Level of Incidence: 2c.

## Authors’ contributions

SJ: data collection, draft of the manuscript. CG: draft of the manuscript, final corrections. GH: clinical FU, regular FU visits, surgical treatment. GS: idea, data collection, statistical analysis, draft of the manuscript, final corrections. All authors were involved in the treatment of the included patients. All authors read and approved the final manuscript.
